# Delivering cytokine mRNA to secondary lymphoid organs for robust cancer immunotherapy

**DOI:** 10.21203/rs.3.rs-8097564/v1

**Published:** 2026-03-25

**Authors:** Tom Anbergen, Yuri van Elsas, Stijn R.J. Hofstraat, Merel M.A. Hendrikx, Jeroen Deckers, Iris Versteeg, Gijs W.B. Ros, Myrthe H.A.P Kapteijns, Branca M. Bartelet, Robby Zwolsman, Mirre M. Trines, Daniek Hoorn, Scott Bex, Pepijn van Houten, Athanasios Ziogas, Cristina Grao-Roldán, Giulia M. Davighi, William Wang, Georgios Soultanidis, Ali Salehi Farid, Yoon Ho Lee, Noortje Voeten, Jurgen M.J. Piek, Gerben M. Franssen, Sandra Heskamp, Henk M. Janssen, P. Michel Fransen, Mireille D Langouo-Fontsa, Andrea Carlo Maria Garavello, Panagiotis Karras, Romana T. Netea-Maier, Carlos Pérez-Medina, Zahi A. Fayad, Mandy M.T. van Leent, Abraham J.P. Teunissen, Mohammad Rashidian, Ewelina Kluza, Yohana C. Toner, Bram Priem, Joost H.C.M. Kreijtz, Thijs J. Beldman, Mihai G. Netea, Roy van der Meel, Willem J.M. Mulder

**Affiliations:** 1 Department of Internal Medicine and Radboud Center for Infectious Diseases (RCI), Radboud University Medical Center, Nijmegen, the Netherlands.; 2 Laboratory of Chemical Biology, Department of Biomedical Engineering, Eindhoven University of Technology, Eindhoven, the Netherlands.; 3 Biotrip B.V., Eindhoven, the Netherlands.; 4 Centro Nacional de Investigaciones Cardiovasculares (CNIC), Madrid, Spain; 5 BioMedical Engineering and Imaging Institute, Icahn School of Medicine at Mount Sinai, New York, NY, USA.; 6 Department of Cancer Immunology and Virology, Dana-Farber Cancer Institute, Boston, MA, USA; 7 Department of Obstetrics and Gynecology, Catharina Cancer Institute, Catharina Hospital, Eindhoven, the Netherlands; 8 Department of Medical Imaging, Nuclear Medicine, Radboud University Medical Center, Nijmegen, the Netherlands; 9 SyMO-Chem B.V., Eindhoven, the Netherlands.; 10 Cardiovascular Research Institute, Icahn School of Medicine at Mount Sinai, New York, NY, USA.; 11 Icahn Genomics Institute, Icahn School of Medicine at Mount Sinai, New York, NY, USA.; 12 Department of Radiology, Brigham and Women’s Hospital, Harvard Medical School, Boston, MA, USA; 13 Molecular Immunology Laboratory, Institut Jules Bordet, Université Libre de Bruxelles, Brussels, Belgium.; 14 Cancer Ecosystems and Metastasis laboratory, Université libre de Bruxelles, Institut Jules Bordet, Brussels, Belgium; 15 Department of Internal Medicine, Division of Endocrinology, Radboud University Medical Center, Nijmegen, The Netherlands; 16 Department of Immunology and Metabolism, Life and Medical Sciences Institute, University of Bonn, Bonn, Germany; 17 These authors contributed equally: T.A., Y.v.E., S.R.J.H., M.M.A.H., J.D.

## Abstract

Recombinant cytokine therapeutics, among the earliest clinical immunotherapies, have yet to reach their full potential despite bioengineering advances. Here, we propose a novel approach focusing on in vivo cytokine production by immune cells in lymphoid organs to mimic these proteins’ physiological function. We developed apolipoprotein nanoparticles (aNPs) to deliver mRNA encoding cytokines, such as interleukin-2 (IL-2), to monocytes in secondary lymphoid tissues. To this end, we engineered a scalable manufacturing process that yields stable aNP-mRNA formulations suitable for long-term storage. Intravenous aNP-mRNA_IL-2_ administration in mice led to sustained IL-2 production in the spleen and lymph nodes, significantly inhibiting tumor growth in B16F10 melanoma and MC38 colon cancer models. We confirmed translational potential through clinical positron emission tomography (PET) imaging and functional cytokine production in non-human primates, and validated aNP-mRNA efficacy on primary immune cells from cancer patients. This mRNA-based cytokine delivery to lymphoid organs offers a promising strategy to enhance cytokine immunotherapy for cancer and potentially other immune-mediated diseases, including autoimmunity, cardiovascular disease, and neurodegenerative conditions.

The first cytokine-based immunotherapy to receive regulatory approval was interferon alpha (IFN-α), authorized by the FDA in 1986 for hairy-cell leukemia^1^. Today, PEGylated IFN-α formulations are applied for the treatment of a variety of malignancies, including certain leukemias^2^, myeloproliferative neoplasms^3^, and melanoma^4^. Although immune checkpoint inhibitors have largely supplanted IFN-α, it retains clinical relevance due to its capacity to promote anti-tumor immunity and increase MHC class I expression^2,3,5^. Interleukin-2 (IL-2) remains the only other recombinant cytokine used in clinical practice. Recombinant IL-2 stimulates cytotoxic T cell activation and proliferation^6^, but its widespread application in humans is limited by severe adverse effects, including vascular leak syndrome and cardiotoxicity^7^. Consequently, IL-2 (aldesleukin) is currently FDA-approved only for metastatic renal cell carcinoma and metastatic melanoma^8^.

Several cytokines are under investigation in clinical trials for cancer and other immune-related disorders, often with modifications to improve efficacy and reduce toxicity. Among recombinant cytokines that are evaluated in cancer trials are interleukin-15 (IL-15)^9^, interleukin-12 (IL-12)^10^, interferon-gamma (IFN-γ)^11^, and granulocyte-macrophage colony-stimulating factor (GM-CSF)^12,13^. Despite being potent immune modulators, cytokine therapies’ unfavorable safety profile and poor bioavailability hampers their development into clinically successful drugs. Bioengineering efforts are therefore focused on improving cytokines’ pharmacokinetic and safety profile, and increasing bioavailability through PEGylation^14^, the development of immunocytokines^15^, and using mRNA-based delivery^16,17^.

Here, we introduce a strategy to deliver mRNA encoding cytokines to secondary lymphoid organs, i.e., spleen and lymph nodes, using proprietary apolipoprotein nanoparticles (aNPs)^18^. We hypothesize that regulating cytokine production in secondary lymphoid organs has immunotherapeutic potential, while minimizing adverse effects observed with systemic treatments. Following aNP formulation optimization, we studied biodistribution using radioactivity-based methods – γ-counting and *in vivo* positron emission tomography (PET) imaging – and assessed functional mRNA translation in mice. We then investigated the anti-tumor efficacy of various intravenous administration regimens using aNPs carrying mRNA encoding human IL-2 (aNP-mRNA_IL-2_) in multiple murine tumor models. To evaluate the translational potential of *in vivo* cytokine therapy using the aNP-mRNA, we also preclinically evaluated an aNP containing mRNA coding for IFN-γ, performed *in vivo* mRNA translation and biodistribution experiments in non-human primates (NHPs) using clinical PET imaging, developed a long-term storing and upscaling strategy, and tested aNP-mRNA formulations on primary immune cells from cancer patients.

## [Results & Discussion]

### Intravenous aNP administration enables mRNA translation in splenic monocytes

Lipid nanoparticles (LNPs) have demonstrated in vivo mRNA translation in hepatocytes, enabling systemic cytokine exposure^19^. Physiologically, cytokines are primarily produced by immune cells in micromilieus, such as lymph nodes, spleen and bone marrow niches, to mobilize specific immune responses^20,21,22^.

**Figure 1.**
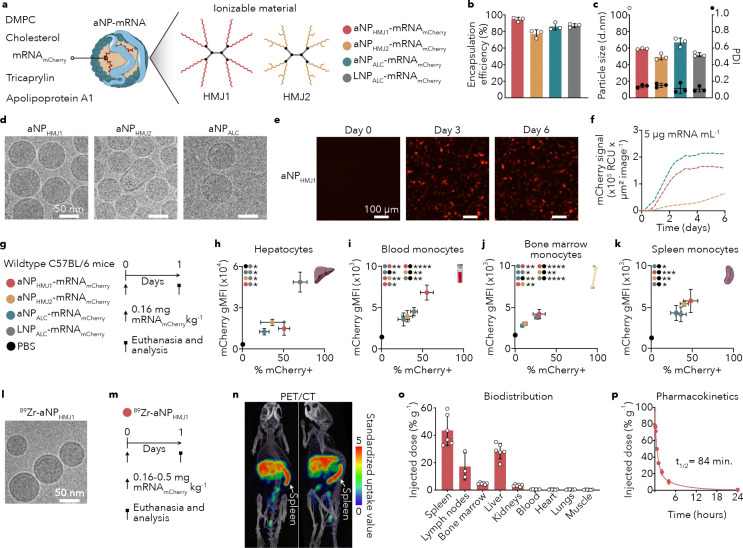
Intravenous aNP administration enables mRNA translation in splenic monocytes **a**, Schematic representation of a mCherry mRNA apolipoprotein nanoparticle (aNP-mRNA_mCherry_) with HMJ1 or HMJ2 ionizable dendrimers. **b**, mRNA encapsulation efficiency (*n* = 3 batches per formulation). **c**, Particle size measured as hydrodynamic diameter and expressed as the number mean average (white dots) and associated poly dispersity index (PDI) (black dots) (*n* = 3 batches per formulation). **d**, Cryo-EM images of aNP_HMJ1_-mRNA_mCherry_, aNP_HMJ2_-mRNA_mCherry_, and aNP_ALC-0315_-mRNA_mCherry_. **e**, Representative live-cell microscopy images acquired 0, 3, and 6 days after transfection. Bone marrow–derived macrophages were transfected for 1 h with 5 μg mRNA_mCherry_/mL, after which the medium was refreshed. **f**, Quantification of mCherry fluorescence signal over time in cells transfected with aNPHMJ1-mRNAmCherry, aNPHMJ2-mRNAmCherry, and aNPALC-0315-mRNAmCherry. Data shown are derived from live-cell imaging and expressed as total red object integrated intensity (relative calibration units (RCU) × μm^2^ / image). **g**, Schematic workflow of intravenously administering aNP-mRNA_mCherry_ and LNP-mRNA_mCherry_ at a dose of 0.16 mg mRNA_mCherry_/kg. **h-k**, mCherry geometric mean fluorescent intensity (gMFI) in hepatocytes (**h**), blood monocytes (**i**), bone marrow monocytes (**j**), and spleen monocytes (**k**) (Y-axis) and percentage of mCherry positive cells (X-axis) 24 hours after intravenous administrating aNP-mRNA_mCherry_ and LNP-mRNA_mCherry_. Data represent mean ± s.d. of one experiment (*n* = 3 mice) and were analyzed by one-way ANOVA with a Bonferroni post-hoc test. Statistically significant differences in gMFI are indicated in the upper left corner by: *P ≤ 0.05, **P ≤ 0.01, ***P ≤ 0.001, ****P ≤ 0.0001. **l**, Cryo-EM of ^89^Zr radiolabeled aNP_HMJ1_-mRNA_mCherry_. **m**, Schematic workflow of mice receiving intravenous ^89^Zr-aNP_HMJ1_-mRNA_mCherry_ (0.16–0.5 mg mRNA/kg, 8–25 μCi) to determine biodistribution and pharmacokinetics. **n**, Positron emission tomography-computed tomography (PET/CT) imaging of mice 24 hours after intravenous administrating ^89^Z-aNP_HMJ1_-mRNA_mCherry_ (25 μCi per mouse). **o**, Quantitative biodistribution of ^89^Z-aNP_HMJ1_-mRNA_mCherry_ by *ex vivo* γ-counting (*n* = 3–6 mice, 8 μCi per mouse). **p**, ^89^Z-aNP_HMJ1_-mRNA_mCherry_ blood pharmacokinetics fitted with a biexponential decay function (*n* = 3 mice, 8 μCi per mouse).

Previously, we introduced the aNP platform for efficient mRNA delivery to bone marrow myeloid cells^18,23^. Here, we developed formulations with lymphoid organ tropism, containing proprietary, multivalent, ionizable cationic molecules, resulting in high-quality formulations – aNP_HMJ1_ and aNP_HMJ2_ – with up to 94% encapsulation efficiency (Fig. 1a). This is in line with the LNP benchmark formulation (LNP_ALC_) and an aNP formulation containing a commercial ionizable molecule ALC-0315 (aNP_ALC_) (87% and 86% respectively, Fig. 1b). **Supplementary Fig. 1a** and **Supplementary Table 1** present details of the production process, the different components’ chemical structures and aNP compositions. Particle sizes of all formulations were approximately 60 nm with narrow size distributions, as established by dynamic light scattering (DLS, Fig. 1c). Cryogenic electron microscopy (cryo-EM) revealed that aNPs_HMJ1_ and aNPs_ALC_ formed homogenous, spherical aNPs, while aNPs_HMJ2_ were less homogenous in shape (Fig. 1d, **Supplementary Fig. 1a**). To confirm that translation occurs in antigen-presenting cells, we evaluated aNP functionality in primary bone marrow-derived macrophages from C57BL/6 mice *in vitro*. We integrated mCherry mRNA (mRNA_mCherry_) and used a live-cell analysis system to measure fluorescence intensity over time (Fig. 1e, **Supplementary Fig. 1b**). After an incubation of one hour and washing cell medium, we measured functional mCherry translation for 6 days for the three different aNP-mRNA_mCherry_ formulations (Fig. 1f).

Following the physicochemical analyses and *in vitro* experiments, we intravenously administered a single dose of either of the three different aNP-mRNA_mCherry_ formulations to C57BL/6 mice, and evaluated their performance against our benchmark LNP formulation (Fig. 1g). In line with previous reports, LNPs enabled functional mRNA translation by hepatocytes, while the three aNP formulations produced significantly less mCherry in these cells (*P*=0.0322 aNP_HMJ1_, *P*=0.0187 aNP_HMJ2_, *P*=0.0147 aNP_ALC_) (Fig. 1h). Conversely, mCherry expression in blood, spleen and bone marrow monocytes (Fig. 1i, j, k) for aNPs_HMJ1_ was elevated compared to LNPs_ALC_. Selected based on its favorable physicochemical properties and desired mRNA translation features in lymphoid organs, we radiolabeled aNP_HMJ1_ with zirconium-89 (^89^Zr-aNP_HMJ1_) to quantitatively study its biodistribution. Cryo-EM revealed that radiolabeling (see **Supplementary Fig. 1c** for details) did not affect aNP morphology and size (Fig. 1l). C57BL/6 (n=6) mice received a single intravenous ^89^Zr-aNP_HMJ1_ dose (0.16–0.5 mg mRNA/kg, 8–25 μCi) (Fig. 1m). *In vivo* PET at 24 hours post intravenous administration revealed high ^89^Zr-aNP_HMJ1_ spleen accumulation (Fig. 1n), which we corroborated *ex vivo* using quantitative γ-counting on excised tissues and organs (Fig. 1o). Besides accumulation in the spleen, we also measured considerable uptake in lymph nodes and determined an ^89^Zr-aNP_HMJ1_ weighted blood half-life of 84 minutes (Fig. 1p). Collectively, the data demonstrate that aNPs_HMJ1_ are spherical and distribute to secondary lymphoid organs and efficiently translated mRNA_mCherry_ in monocytes upon intravenous administration.

### Interleukin-2 production in secondary lymphoid organs drives effective and safe tumor growth inhibition

**Figure 2.**
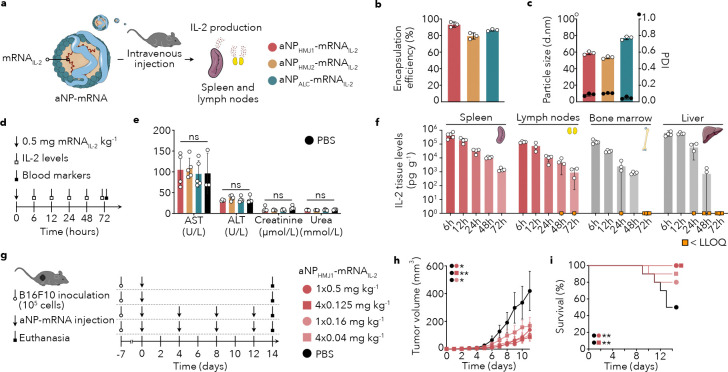
Interleukin-2 production in secondary lymphoid organs drives effective and safe tumor growth inhibition **a**, Schematic representation of a human IL-2 mRNA (mRNA_IL-2_) containing aNP being administered intravenously and inducing IL-2 production in secondary lymphoid organs. **b**, mRNA encapsulation efficiency of aNPs-mRNA_IL-2_ (*n* = 3 batches per formulation). **c**, Particle size measured as hydrodynamic diameter and expressed as the number mean average (white dots) and associated poly dispersity index (PDI) (black dots) (*n* = 3 batches per formulation). **d**, Schematic showing workflow to determine IL-2 levels at 6, 12, 24, 48, and 72 hours after treatment. Additionally, after 72 hours blood markers were measured. **e**, Blood chemistry indicating aNP-mRNA’s biocompatibility, as measured by aspartate aminotransferase (AST), alanine transferase (ALT), creatinine and urea following 72 hours after a single intravenous administration of 0.5 mg mRNA_IL-2_/kg. Data represent mean ± s.d. of one experiment (*n* = 3–5 mice) and analyzed by one-way ANOVA with a Bonferroni post-hoc test. **f**, Organ IL-2 levels in the spleen, lymph nodes, bone marrow, and liver determined 6, 12, 24, 48, and 72 hours following intravenous administration of 0.5 mg mRNA_IL-2_/kg with aNP_HMJ1_-mRNA_IL-2_. Data represent mean ± s.d. of one experiment (*n* = 3–5 mice). **g**, Schematic treatment regimen involving B16F10-tumor bearing mice (*n* = 10–11 mice) receiving either single or repeated intravenous administration of aNP_HMJ1_-mRNA_IL-2_ with a cumulative mRNA_IL-2_ dose of 0.5 mg/kg (1× 0.5 mg/kg and 4× 0.125 mg/kg) or 0.16 mg/kg (1× 0.16 mg/kg and 4× 0.04 mg/kg). **h**, Tumor growth curve of C57BL/6 mice inoculated with 1×10^5^ B16F10 cells and treated with either PBS (black) or aNP_HMJ1_-mRNA_IL-2_ (red). Data represent mean ± s.e. of one experiment (*n* = 10–11 mice). Tumor growth rate was analyzed by one-way ANOVA with a Bonferroni post-hoc test. Statistically significant differences between tumor growth rates are indicated in the upper left corner by: *P ≤ 0.05, **P ≤ 0.01. **i**, Survival curve of B16F10 tumor-bearing mice (*n* = 10–11 mice) after treatment with either PBS (black) or aNP_HMJ1_-mRNA_IL-2_ (red). Data were analyzed using the log-rank (Mantel-Cox) test and statistically significant differences are indicated by: **P ≤ 0.01.

IL-2 is a central regulator of cytotoxic T cell activation ^24^. Under physiological conditions, CD4^+^ T cells secrete IL-2 in secondary lymphoid organs to coordinate antigen-specific immunity ^24–26^. It is well-established that human IL-2 stimulates T cell activity in mice^27^. Following integration of mRNA_IL-2_, we designed experiments to evaluate the three different aNPs formulations’ ability to produce human IL-2 in the spleen and lymph nodes in mice *in vivo* (Fig. 2a). With mRNA encapsulation efficiencies of up to 94% (Fig 2b), sizes ranging from 50 to 80 nm, and polydispersities lower than 0.1 (Fig. 2c), the aNPs containing mRNA_IL-2_ were very similar to those containing mRNA_mCherry_ (Fig. 1b, c). A single intravenous dose in mice of any of the three aNP-mRNA_IL-2_ formulations was well tolerated and produced markedly different IL-2 serum levels over time compared to recombinant IL-2 (Fig. 2d, **Supplementary Fig. 2a**). Blood chemistry parameters revealed no significant differences in liver and kidney markers of mice that received aNP-mRNA_IL-2_ as compared to PBS (Fig. 2e).

Informed by its favorable safety profile, mRNA translation potential in secondary lymphoid organs and IL-2 production efficacy, we selected the aNP_HMJ1_ for subsequent experiments. We first longitudinally studied IL-2 concentration in lymphoid organs and the liver following a single aNP-mRNA_IL-2_ dose (Fig. 2d). ELISA detected IL-2 concentrations in the spleen, lymph nodes, and bone marrow, ranging from 3× 10^4^ to 5× 10^5^ pg/g tissue (Fig. 2f). By 72 hours, IL-157 2 was largely undetectable in the bone marrow and liver.

After establishing that a single intravenous aNP_HMJ1_-mRNA_IL-2_ dose resulted in sustained IL-2 levels in the spleen and lymph nodes, we used the B16F10 mouse melanoma model in a first therapeutic *in vivo* experiment. Mice inoculated with B16F10 tumor cells are a model for a highly immunosuppressive and fast-growing cancer. Because the B16F10 melanoma model is resistant to checkpoint blockade^28^, efficacy in this setting underscores the potency of localized cytokine production^28^. We designed an experiment to evaluate aNP_HMJ1_-mRNA_IL-2_ treatment in mice bearing B16F10 tumors at two dose levels, given as a single intravenous injection or as four injections every four days (Fig. 2g). Tumor growth profiling revealed that both dose levels and the two different injection schemes significantly inhibited tumor growth for 1× 0.5 mg/kg (*P*=0.0421), 4× 0.125 mg/kg (*P*=0.0016) and 1× 0.16 mg/kg (*P*=0.0262) aNP_HMJ1_-mRNA_IL-2_ compared to PBS (Fig. 2h), and improved overall survival (Fig. 2i).

### Intravenous aNP treatment induces CD8^+^ T cell expansion in tumor-bearing mice

**Figure 3.**
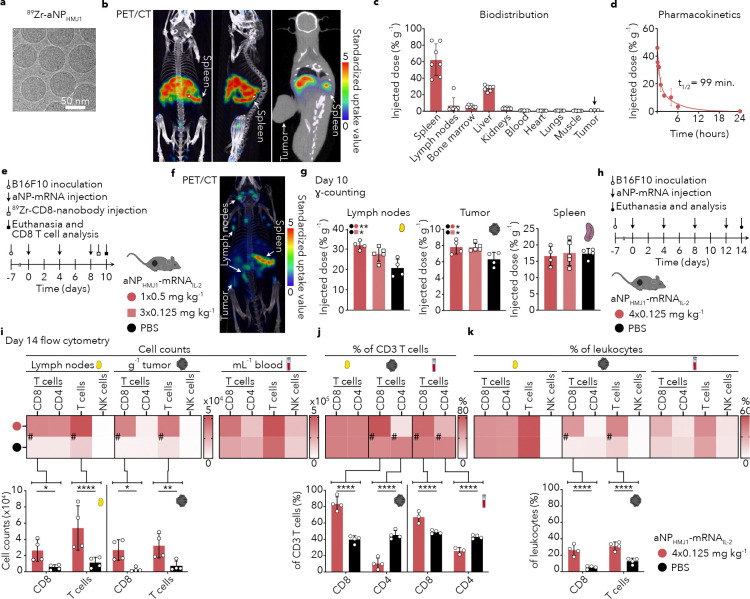
Intravenous aNP treatment induces CD8 T cell expansion in tumor-bearing mice **a**, ^89^Zr radiolabeled aNP_HMJ1_-mRNA_IL-2_ cryo-EM image. **b**, PET/CT imaging of mice 24 hours after intravenous administration of ^89^Zr-aNP_HMJ1_-mRNA_IL-2_ (0.5 mg mRNA_IL-2_/kg, 25 μCi per mouse). **c**, Quantitative biodistribution of ^89^Zr-aNP_HMJ1_-mRNA_IL-2_ by ex vivo γ-counting (n = 3–7 mice, 0.16 mg mRNA_IL-2_/kg, 8 μCi per mouse). **d**, ^89^Zr-aNP_HMJ1_-mRNA_IL-2_ blood pharmacokinetics fitted with a biexponential decay function (n = 7 mice, 0.16 mg mRNA_IL-2_/kg, 8 μCi per mouse). **e**, Schematic treatment regimen involving B16F10-tumor bearing mice receiving PBS (black) and either single or repeated intravenous administration of aNP_HMJ1_-mRNA_IL-2_ (red) with a mRNA dose of 1× 0.5 mg/kg (circle) and 3× 0.125 mg/kg (square), and a single intravenous administration of ^89^Zr-CD8-nanobody. **f**, Positron emission tomography-computed tomography (PET/CT imaging of mice 24 hours after intravenous administration of ^89^Zr-CD8-nanobody (16 μCi per mouse) on day 10. **g**, Quantitative biodistribution of ^89^Zr-CD8-nanobody by ex vivo γ-counting (n = 4–5 mice, 16 μCi per mouse) in lymph nodes, spleen, and tumor on day 10. Data represent mean ± s.d. of one experiment (*n* = 4–5 mice) and analyzed by one-way ANOVA with a Bonferroni post-hoc test. Statistically significant differences in injected dose (%ID g^−1^) are indicated in the upper left corner using *P ≤ 0.05 and **P ≤ 0.01, **h**, Schematic of our treatment regimen involving B16F10-tumor bearing mice receiving PBS (black) or repeated intravenous administration of aNP_HMJ1_-mRNA_IL-2_ with a cumulative mRNA dose of 0.5 mg/kg (4× 0.125 mg/kg) (red). **i-k,** Flow cytometry results of lymphoid cell subsets count **(i),** normalized to two lymph nodes, per gram of tumor, and per mL of blood, percentage of CD8^+^ T cells and CD4^+^ T within the total T cell (CD3^+^) population **(j),** and lymphoid cell subsets as percentage of total leukocytes **(k)** in lymph nodes, tumor, and blood, presented as heatmaps and bar graphs. Data represent mean ± s.d. of one experiment (*n* = 4 mice) and analyzed by two-way ANOVA with a Bonferroni post-hoc test. Statistically significant differences are indicated by: *P ≤ 0.05, **P ≤ 0.01, ****P ≤ 0.0001.

To study immunological responses longitudinally following a single intravenous dose, we first studied aNP-mRNA_IL-2_’s pharmacokinetic profile using ^89^Zr radiolabeling. The labeling did not affect particle morphology and physicochemical properties (Fig. 3a, **Supplementary Fig. 3a**). Similar to the biodistribution data presented in Figs. 1l–p for aNP_HMJ1_-mRNA_mCherry_, *in vivo* PET imaging revealed that aNP_HMJ1_-mRNA_IL-2_ strongly accumulated in the spleen, and not in the tumor (Fig. 3b). *Ex vivo* γ-counting revealed a two-fold higher concentration in the spleen as compared to the liver (Fig. 3c) and a weighted blood half-life of 99 minutes (Fig. 3d).

Ten days following intravenous aNP_HMJ1_-mRNA_IL-2_ administration, we first immunologically profiled cytotoxic T cell expansion using a CD8-specific radiotracer. The CD8 probe consists of a nanobody that is radiolabeled with ^89^Zr, allowing quantitatively studying cytotoxic T cell dynamics at the whole mouse level, as we have reported previously^29^. *In vivo* PET imaging revealed high levels of CD8^+^ T cells in the spleen, lymph nodes and tumor in mice that received intravenous aNP_HMJ1_-mRNA_IL-2_ treatment (Fig. 3e, f). *Ex vivo* γ-counting measured significantly higher CD8^+^ T cell accumulation in both the lymph nodes and tumors of aNP_HMJ1_-mRNA_IL-2_ treated mice as compared to PBS (Fig. 3g), indicative of cytotoxic T cell expansion and migration from lymph nodes to the tumor. We therefore used flow cytometry to profile the immune landscape of the lymph nodes and tumor 14 days after initiating aNP_HMJ1_-mRNA_IL-2_ treatment (Fig. 3h). We found consistently higher numbers of CD8^+^ T cells in both lymph nodes and tumor, while NK cell numbers did not increase (Fig. 3i). These immune landscape changes are evident from the relative representation of CD8^+^ and CD4^+^ within the CD3^+^ T cell (Fig. 3j) or leukocyte (Fig. 3k) compartment. Although the total T cell count increased following aNP_HMJ1_-mRNA_IL-2_ treatment, both measures showed unchanged CD8^+^/CD4^+^ ratio in lymph nodes, but we did observe this ratio to be significantly increased in tumors (*P*<0.0001), indicating a cytotoxic T cell-mediated anti-tumor response.

### Biodistribution and functional mRNA translation in non-human primates

**Figure 4.**
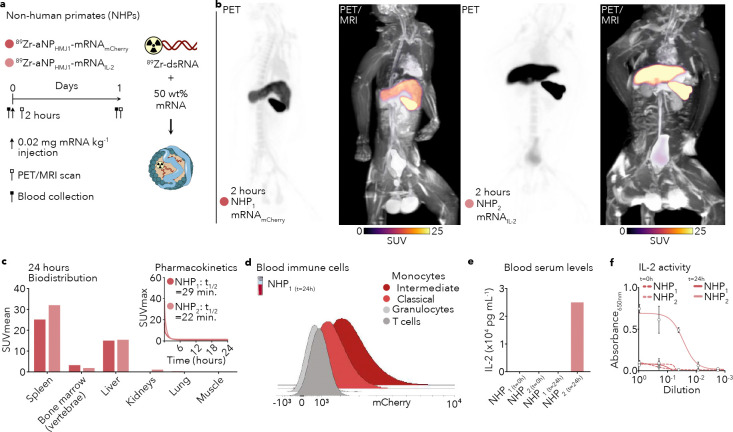
Biodistribution and functional mRNA translation in non-human primates (NHPs) **a**, Schematic workflow of intravenously administering ^89^Zr-aNP_HMJ1_-mRNA_mCherry_ (0.95 mCi per NHP_1_) and ^89^Zr-aNP_HMJ1_-mRNA_IL-2_ (1.78 mCi per NHP_2_) at a dose of 0.02 mg mRNA/kg with 0.02 mg ^89^Zr-dsRNA/kg in cynomolgus macaques. **b**, PET-magnetic resonance imaging (PET/MRI) of NHPs 2 hours after intravenous administration. **c**, Biodistribution expressed as mean standardized uptake values (SUV_mean_) 24 hours after intravenous administration and blood pharmacokinetics expressed as maximum standardized uptake values (SUV_max_) of the left ventricle blood pool fitted with a biexponential decay function. **d,** Histograms of mCherry fluorescence intensity in myeloid and lymphoid cell subsets in the blood 24 hours after intravenous administration of ^89^Zr-aNP_HMJ1_-mRNA_mCherry_ in NHP_1_. **e**, Serum IL-2 concentrations measured at baseline (t=0h, before injections) and 24 hours (t=24h) after intravenous administration of ^89^Zr-aNP_HMJ1_-mRNA_mCherry_ (NHP_1_) and ^89^Zr-aNP_HMJ1_-mRNA_IL-2_ (NHP_2_). **f**, IL-2 activity measured by HEK-Blue IL-2 reporter cells, quantified via secreted embryonic alkaline phosphatase (SEAP) colorimetric readout (absorbance at 650 nm).

After demonstrating aNP-mRNA efficacy and identifying the mechanism by which it exerts anti-cancer immunity, we studied its translational potential in non-human primates (NHPs). Two cynomolgus macaques were included to evaluate blood half-life and biodistribution by *in vivo* PET combined with magnetic resonance imaging (PET/MRI), and mRNA translation after receiving a single dose of either ^89^Zr-labeled aNP_HMJ1_-mRNA_mCherry_ or aNP_HMJ1_-mRNA_IL-2_ (Fig. 4a; PET/MRI scans after 2 hours Fig. 4b). PET/MRI analyses standardized uptake values (SUVs) revealed that the pharmacokinetic profile of aNP_HMJ1_-mRNA_mCherry_ and aNP_HMJ1_-mRNA_IL-2_ were very similar, with blood half-lives of 29 and 22 minutes, respectively (Fig. 4c). At 24 hours post intravenous administration, spleen accumulation was approximately 1.5-fold higher than liver accumulation for both formulations (Fig. 4c). Importantly, flow cytometry on whole blood samples showed functional mRNA translation of mCherry in monocytes at 24 hours following intravenous aNP_HMJ1_-mRNA_mCherry_ (Fig. 4d). In line with this observation, we measured 2.5× 10^4^ pg/mL IL-2 at 24 hours following a single intravenous aNP_HMJ1_-mRNA_IL-2_ dose (Fig. 4e). It is important to note that we did not detect IL-2 in the serum before aNP-mRNA injections or in the aNP_HMJ1_-mRNA_mCherry_ injected NHP_1_, demonstrating that IL-2 production was driven by functional mRNA_IL-2_ translation, and not due to an immunological response to aNP administration. Finally, we collected serum and established that IL-2 produced by an NHP following intravenous aNP_HMJ1_-mRNA_IL-2_ treatment was functional using a SEAP reporter cell line (Fig. 4f), which is typically used to determine this cytokine’s activity^30^.

### Reproducible aNP manufacturing and long-term storing strategy

**Figure 5.**
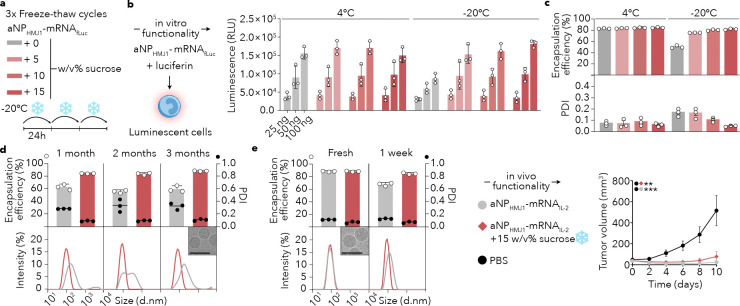
Reproducible aNP manufacturing and long-term storing strategy **a**, aNP_HMJ1_-mRNA_fLuc_ were formulated with varying sucrose concentrations for cryoprotection and evaluated after three freeze–thaw cycles (−20 °C) or storage at 4°C or −20 °C (*n* = 3 batches per formulation). **b**, In vitro functionality defined by luminescence (Relative Light Units (RLU)) 24h after transfection of RAW264.7 cells using three different doses (25ng, 50ng and 100ng mRNA). **c**, mRNA encapsulation efficiency, and PDI of aNP_HMJ1_-mRNA_fLuc_ after three freeze-thaw cycles. **d**, Stability of aNP_HMJ1_-mRNA_fLuc_ at −20 °C cryoprotected with 0 w/v% or 15 w/v% sucrose over 3 months of storage. Encapsulation efficiency (white dots, left Y-axis) and PDI (black dots, right Y-axis) are shown at indicated timepoints. Particle size distributions are displayed as relative light scattering intensity vs. hydrodynamic diameter (d.nm). Cryo-EM images show aNPs formulated after 3 months at −20°C cryoprotected with 15 w/v% sucrose. **e,** Tumor growth curve of C57BL/6 mice inoculated with 1×10^6^ MC38 cells and treated with a single 0.5 mg mRNA_IL-2_ /kg injection of either freshly formulated aNP_HMJ1_-mRNA_IL-2_ (grey), aNP_HMJ1_-mRNA_IL-2_ stored for one week at −20°C with 15 w/v% sucrose (red), or PBS (black). Data represent mean ± s.e. of one experiment (*n* = 15 mice). Tumor volume at day 14 was analyzed by one-way ANOVA with a Bonferroni post-hoc test. Statistically significant differences in tumor volume are indicated in the upper left corner by: **P ≤ 0.01, ***P ≤ 0.001.

Establishing and controlling chemistry, manufacturing and controls (CMC) is essential for clinical translation. Manufacturing of a therapeutic must be rigorous, scalable, and reproducible^31,32^. As part of CMC efforts, forced degradation (**Supplementary Fig. 4a**) stability studies are essential to ensure a therapeutic’s robustness. To further study the aNP platform’s translational potential, we designed a CMC workflow that focused on cryoprotection, studying long-term storage conditions and batch-to-batch reproducibility of an aNP formulation containing luciferase mRNA (mRNA_fLuc_, Fig. 5a, **Supplementary Fig. 4b**). The CMC effort revealed that reconstituting in 5%, 10% or 15% sucrose allowed three freeze-thaw cycles without compromising *in vitro* mRNA translation efficacy (Fig. 5b). At lower sucrose percentages of 5% and 10%, aNP polydispersity increased from 0.1 to 0.2, indicative of nanoparticle aggregation (Fig. 5c). Cryoprotection using 15% sucrose enabled long-term storage while preserving aNP physicochemical characteristics up to three months at −20 °C (Fig. 5d). As an ultimate test, we cryoprotected aNP_HMJ1_-mRNA_IL-2_ and stored the batch at −20 °C for a week. After thawing the batch, a single administration of aNP_HMJ1_-mRNA_IL-2_ reduced tumor growth in mice bearing subcutaneous MC38 colon tumors to levels comparable to those of a freshly prepared batch (Fig. 5e).

### Cytokine mRNA delivery using aNPs is a versatile anti-tumor treatment modality

**Figure 6.**
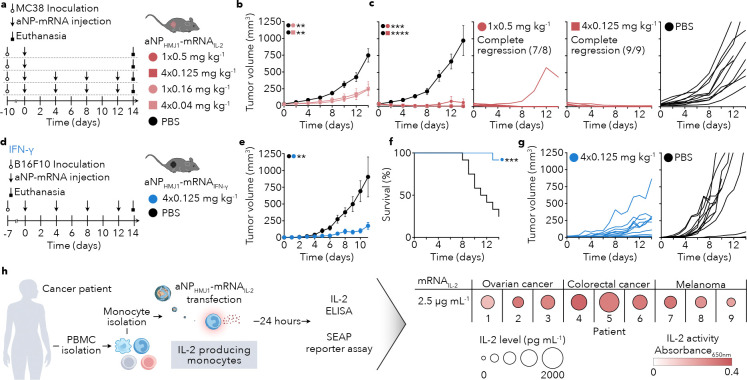
Cytokine mRNA delivery using aNPs is a versatile anti-tumor treatment modality **a**, Schematic treatment regimen involving MC38 tumor bearing mice (*n* = 6–10 mice) receiving either single or repeated intravenous administration of aNP_HMJ1_-mRNA_IL-2_ with a cumulative mRNA dose of 0.5 mg/kg (1× 0.5 mg/kg; 4× 0.125 mg/kg) or 0.16 mg/kg (1× 0.16 mg/kg; 4× 0.04 mg/kg). **b**,**c,** Tumor growth curves and individual tumor growth curves of C57BL/6 mice inoculated with 1×10^6^ MC38 cells and treated with either PBS (black) or aNP_HMJ1_-mRNA_IL-2_ (red). Data represent mean ± s.e. of one experiment (*n* = 6–10 mice). Tumor volume at day 14 was analyzed by one-way ANOVA with a Bonferroni post-hoc test. Statistically significant differences in tumor volume are indicated in the upper left corner by: **P ≤ 0.01, ***P ≤ 0.001, ****P ≤ 0.0001. **d**, Schematic treatment regimen involving B16F10-tumor bearing mice (*n* = 11–13 mice) receiving four intravenous administrations of 0.125 mg/kg aNP_HMJ1_-mRNA_IFN-γ_ every four days. **e**, Tumor growth curve of C57BL/6 mice inoculated with 1×10^5^ B16F10 cells and treated with either PBS (black) or aNP_HMJ1_-mRNA_IFN-γ_ (blue). Data represent mean ± s.e. of one experiment (*n* = 11–13 mice). Tumor growth rate was analyzed by an unpaired t-test. Statistically significant differences in tumor growth rate are indicated in the upper left corner by: **P ≤ 0.01. **f**, Survival curve of B16F10 tumor-bearing mice (*n* = 11–13 mice) after treatment with either PBS (black) or aNP_HMJ1_-mRNA_IFN-γ_ (blue). Data are analyzed with log-rank (Mantel-Cox) test and statistically significant differences are indicated by: ***P ≤ 0.001. **g**, Individual tumor growth curves of B16F10 tumor-bearing mice treated with either aNP_HMJ1_-mRNA_IFN-γ_ (blue, left) or PBS (black, right). **h**, Schematic overview of human monocyte transfection with aNP_HMJ1_-mRNA_IL-2._ Primary monocytes were isolated from PBMCs of nine cancer patients (ovarian, colorectal, melanoma) and transfected with 2.5 μg mRNA_IL-2_/mL. IL-2 secretion was quantified by ELISA and IL-2 activity was measured by HEK-Blue IL-2 reporter cells, quantified via SEAP colorimetric readout. Results are displayed as a bubble heatmap: circle area encodes IL-2 level (pg/mL) and color scale encodes IL-2 activity (absorbance at 650 nm). Each symbol denotes one patient sample.

*In vivo* production of IL-2 using aNPs targeting secondary lymphoid organs is a strategy that holds promise for treating diverse cancers. In the MC38 colon cancer mouse model, a single intravenous aNP_HMJ1_-mRNA_IL-2_ dose of 0.16 mg mRNA/kg or four doses of 0.04 mg mRNA/kg significantly inhibited tumor growth (Fig. 6a, b). At 0.5 mg/kg and four doses of 0.125 mg/kg, we observed complete tumor remission in 7 out of 8 mice and 9 out of 9 mice, respectively (Fig. 6c).

The aNP platform can be readily adopted to deliver mRNA encoding a wide range of cytokines to secondary lymphoid organs. Besides IL-2, which promotes the expansion of cytotoxic CD8 T cells, other cytokines engage different immune cell populations with anti-tumor function. Our aNP-mRNA delivery may also hold potential to mobilize an anti-tumor myeloid response with IFN-γ. We therefore produced aNPs incorporating mRNA encoding for IFN-γ. In the B16F10 model, four intravenous aNP_HMJ1_-mRNA_IFN-γ_ doses (0.125 mg mRNA/kg) (Fig. 6d) significantly inhibited tumor growth (*P*=0.0011) (Fig. 6e) and prolonged overall survival (*P*=0.0006) (Fig. 6f). The individual tumor growth profiles revealed a strong inhibition in 9 out of 10 mice with little variation, indicative of this approach’s consistent efficacy (Fig. 6g).

To further evaluate our strategy’s translational potential, we performed ex vivo experiments on monocytes obtained from colon and ovarian cancer and melanoma patients. Monocytes were isolated from peripheral blood mononuclear cells (PBMCs) and transfected with either aNP_HMJ1_-mRNA_mCherry_ or aNP_HMJ1_-mRNA_IL-2_. We measured functional IL-2 production after 24 hours, while IL-2 was not detected in aNP_HMJ1_-mRNA_mCherry_ transfected monocytes (Fig. 6h, **Supplementary Fig. 5a**).

## Conclusion

Bioengineering efforts this century have largely focused on protein engineering to improve cytokine-based therapeutics’ pharmacokinetic profile and bioavailability. Hepatocyte-directed mRNA delivery via LNPs for producing long-circulating IL-2–albumin fusion proteins has shown potential^16^. In another study, injecting IL-2-encoding circRNA in the tumor periphery also halted tumor growth^33^. Cytokines are secreted by immune cells within specific microenvironments where they are needed to mediate physiological responses. Here, we developed and applied an mRNA delivery strategy – using our proprietary aNP platform – that durably produces cytokines in secondary lymphoid organs as a robust cancer immunotherapy approach. Intravenous administration of aNPs containing mRNA encoding IL-2 or IFN-γ demonstrated biodistribution to lymphoid organs, sustained cytokine production, and significant anti-tumor efficacy across various mouse tumor models. Besides biodistribution and mRNA translation efficacy in mice, PET/MRI experiments showed that aNPs distribute to the spleen and efficiently produce cytokines in NHPs. CMC efforts demonstrated that various aNP-mRNA formulations can be reproducibly manufactured, cryoprotected, and stored at −20 °C for at least three months, highlighting the platform’s translational potential. Beyond its use in cytokine-based immunotherapy for cancer, the aNP platform holds potential for treating autoimmune and inflammatory conditions, including lupus, cardiovascular diseases, inflammatory bowel disease, and neurodegenerative disorders.

## Methods

### Animals

All animal experiments conducted were approved by the Radboud University Medical Center’s Dierexperimentencommissie (DEC) (CCD: AVD10300 2021 15550 and AVD10300 2021 14977) and complied with both European and Dutch guidelines according to the care and use of laboratory mice. 6–8-week-old female C57BL/6J mice were purchased from Charles River Germany and co-housed at ambient temperature (22–24°C) and 45–65% RV humidity level. Mice were randomized and assigned to control and treatment groups. Studies were conducted in a blinded manner. Non-human primate experiments were performed in accordance with Icahn School of Medicine at Mount Sinai Institutional Animal Care and Use Committee (IACUC).

### Patients

For colorectal carcinoma patients, the study was approved by the local Ethics Committee (Commissie Mensgebonden Onderzoek Arnhem-Nijmegen, 2021–13380). For ovarian carcinoma patients, the study was approved by the Medical Research Ethics Committees United, Commissie Mensgebonden Onderzoek, NL number: NL84392.000.23. For melanoma patients, the study was approved by the Ethical Committee of The Institute Jules Bordet (Accreditation number OM011, Eudract number 2021–000027-12, Internal reference CE3272).

Peripheral blood from patients was collected into EDTA-anticoagulated tubes. Blood was then transferred to Falcon tubes and diluted to 35 mL with PBS. Peripheral blood mononuclear cells (PBMCs) were then isolated from blood using Ficoll gradient and SepMate tubes (StemCell Technologies). Cell counts were obtained using Sysmex, and PBMCs were frozen using CryoStor (StemCell Technologies). On the day of the experiment, PBMCs were thawed, cell counts were obtained and monocytes were isolated via magnetic separation using the Pan Monocyte Isolation Kit (Miltenyi Biotec, Cat #130–096-537) following the manufacturer’s instructions. Monocytes were plated (1 × 10^5^/well) into a flat-bottom 96-well plate and rested for 1 h. Then, monocytes were transfected and incubated for 24 h. Following incubation, cell supernatant was harvested for analysis by ELISA and SEAP assay.

### Ionizable cationic material synthesis

HMJ1 was synthesized by Michael-addition acrylate functionalization of a G1-PPI core. G1-PPI-(NH_2_)_4_ (n-butylene core; 200 mg, 0.63 mmol) was dissolved in iso-propanol (2ml) in a sealed reaction tube. n-Octyl acrylate (1.86 g, 10.1 mmol, 16 equiv.) was added and the reaction mixture was heated at 55 °C for 4 days under stirring and an inert nitrogen atmosphere. The reaction progress was monitored by ^1^H-NMR, and after full conversion, the solvent was removed in vacuo. The crude product was purified by analogous work-up to afford HMJ1 as a viscous, slightly yellowish oil (758 mg, 67%). MALDI-TOF-MS (CHCA matrix, positive reflector mode): obs. m/z = (M+H)+ 1791.52, (M+Na)+ 1813.52, (M+K)+ 1829.49; calculated for C_104_H_200_N_6_O_16_: exact mass 1789.50, molecular weight 1790.77. HMJ2 was synthesized by Michael-addition acrylate functionalization of a G1-PAMAM core. A stirred solution of G1-PAMAM-C2 (ethylene-diamine core; 4 amine end groups; 165 mg, 0.32 mmol) in propan-2-ol (2 ml) was placed in a reaction tube, and 2-ethyl-hexylacrylate (589 mg, 3.2 mmol, 10 equiv.) was added. After partial conversion, more 2-ethylhexyl acrylate (600 mg, 3.3 mmol, 10 equiv.) was added. The reaction mixture was heated to 60 °C for 408 h under stirring. After completion, the solvent was removed in vacuo, and the crude product was purified by analogous work-up to yield HMJ2 as a yellow viscous oil (270 mg, 0.13 mmol, 41%). MALDI-TOF-MS (CHCA matrix, positive reflector mode): obs. m/z = (M+H)+ 1991.56, (M+Na)+ 2013.53; calculated for C_110_H_208_N_10_O_20_: exact mass 1989.56, molecular weight 1990.92. ALC-0315 was purchased from SyMO-Chem B.V. The chemical structures are presented in **Supplementary Fig. 1a.**

### mRNA production

For in vitro and mouse studies, sequence-optimized human IL-2, murine IFN-γ and mCherry mRNAs were obtained from OZ Biosciences, and firefly luciferase (fLuc) mRNA was obtained from RiboPro. All mRNAs were produced by in vitro transcription, stabilized with a Cap1 structure at the 5′ end and a poly(A) tail at the 3′ end, and fully substituted with N1-methyl-pseudouridine. For non-human primate studies, high-quality research-grade mRNA was supplied by eTheRNA. During in vitro transcription, all uridine residues were replaced with N1-methyl-pseudouridine, and a 90-adenosine poly(A) tail was co-transcriptionally incorporated. 5′ capping was performed using CleanCap. The resulting mRNA was column-purified and qualified by testing for endotoxin (<1 EU/mg mRNA), residual plasmid DNA, protein impurities (<0.2 μg/mg), and double-stranded RNA. The mRNA sequences are presented in **Supplementary Table 1.**

### aNP-mRNA formulation

aNPs containing ionizable materials using a 6:1 ratio of nitrogen to phosphate (N/P ratio) were generated by rapid T-junction mixing of organic lipid and aqueous mRNA phases. The formulation process, amounts, molar ratios and chemical structures of the components are presented in **Supplementary Fig. 1a** and **Supplementary Table 1**.

For aNP-mRNA preparation, an ethanolic lipid phase containing 1,2-dimyristoyl-sn-glycero-3-phosphocholine (DMPC, 850345; Avanti Polar Lipids), an ionizable material (HMJ1, HMJ2 or ALC-0315; SyMO-Chem B.V.), cholesterol (C8667; Sigma-Aldrich) and tricaprylin (T1978000; Sigma-Aldrich) was prepared to a total volume of 1.17 mL at a molar ratio of 20:3:20:57 (DMPC:HMJ1/HMJ2:cholesterol:tricaprylin) or 17:17:17:49 (DMPC:ALC-0315:cholesterol:tricaprylin) with an N/P ratio of 6. A separate aqueous phase containing 100 μg mRNA in 25 mM sodium acetate (pH 4.0) was prepared in a volume of 3.5 mL organic and aqueous phases were combined at a 1:3 (v/v) organic-to-aqueous flow-rate ratio using rapid T-junction mixing^34^ (with an organic flow-rate of 7 mL/min and an aqueous flow-rate of 21 mL/min) to yield mRNA-loaded aNPs, which were immediately transferred to 12–14 kDa MWCO dialysis membranes (Spectra/Por) and dialyzed against 1× PBS (pH 7.4) overnight (18 hours). The following day, dialyzed aNP-mRNA formulations were adjusted to a final volume of 6 mL, after which 2 mL apolipoprotein A1 (apoA1; 0.26 mg/mL), isolated from HDL plasma fraction (Medix Biochemica), was introduced via T-junction mixing at a 1:3 flow-rate ratio (apoA1 solution: suspension) and incubated for 20 min at room temperature to obtain the aNP-mRNA. Using aseptic technique, the aNP-mRNA suspension was passed through a 0.2 μm sterile filter and concentrated by centrifugal filtration at 1100× g using 100 kDa MWCO Vivaspin filters (Amicon), then adjusted to the desired final mRNA concentration. Unless otherwise specified, formulations were stored at 4 °C until use.

### aNP-mRNA physicochemical analysis

The hydrodynamic diameter (expressed as the number-weighted mean) and polydispersity index (PDI) were determined using a Zetasizer Nano ZSP (Malvern Instruments). Prior to measuring, samples were diluted 6 times in 1x PBS (pH 7.4) and equilibrated at room temperature before the measurement. Each sample was measured 3 times with 10 runs of 10 seconds, without fixing the attenuator and measurement position. The Quant-itTM RiboGreen RNA Assay Kit (Thermo Fisher Scientific) was used to quantify mRNA encapsulation efficiency as previously described^18^. In short, samples were diluted in 1x Tris-EDTA (TE) buffer and TE buffer supplemented with 2% Triton X-100 (TE-T) to a total volume of 100 μL in a black 96well plate. From the corresponding mRNA stock, a standard curve was generated by serial dilution. Next, 200-fold diluted RiboGreen reagent in TE or TE -T was added to bring the total volume to 200 μL. Fluorescence was measured on a Tecan Spark microplate reader, set at 480 nm excitation and 520 nm emission wavelengths. The RNA encapsulation efficiency was calculated as [(Total RNA - Unencapsulated RNA) / Total RNA] * 100.

### Storage and stability of aNP-mRNA

For storage and stability studies, freshly formulated Firefly luciferase (Fluc; RiboPro) mRNAaNPs were diluted in PBS containing sucrose (sterile filtered) in defined ratios to obtain sucrose concentrations of 0, 5, 10, and 15 w/v%. The samples were frozen at −20°C for different periods up to 3 months. For freeze-thaw stability analysis, each sucrose concentration was subjected to three freeze-thaw cycles with each cycle consisting of 24 hours of freezing at −20°C followed by thawing on ice. Physicochemical analyses were performed as described above before freezing and after thawing on ice for each time-point. To test mRNA-aNP functionality *in vitro*, 3×10^4^ RAW264.7 cells were seeded in 96-well plates and transfected with Fluc mRNA-aNPs after each storage time point. After 24 hours, luciferase activity was quantified using the ONE-Glo Luciferase Assay System (Promega) and normalized to corresponding metabolic activity measured by CellTiter 96^®^ AQ_ueous_ One Solution Cell Proliferation Assay (MTS) (Promega), according to the manufacturer’s protocol. All experiments were performed in triplicate.

Following stability testing, 100 representative nanoparticles were randomLy selected from each Cryo-TEM image for all storage conditions. Nanoparticles were first manually annotated in ImageJ (National Institutes of Health, USA) by outlining intact particles. Subsequently, structural artifacts, including blebbing and empty particles, were annotated separately. The cross-sectional area of each nanoparticle and artifact was quantified using ImageJ. These measurements were used to calculate (i) the percentage of nanoparticles exhibiting artifacts and (ii) the percentage of the total nanoparticle surface area occupied by artifacts. For forced degradation studies, freshly formulated fLuc mRNA-aNPs were subjected to different conditions as indicated in **Supplementary Fig. 4a**. Physicochemical and functional analysis was performed as described above.

### Cryogenic transmission electron microscopy (cryo-TEM) of aNP-mRNA

Prior to vitrification, 200-mesh lacey carbon-supported copper grids (Electron Microscopy Sciences) were plasma treated for 40s using a Cressington 208 carbon coater. Next, 3μL of aNP-mRNA sample was applied to each grid and vitrified into a thin film by plunge freezing in liquid ethane using an automated robot (FEI Vitrobot Mark IV). Cryo-EM imaging was conducted on a cryoTITAN transmission electron microscope (Thermo Fisher Scientific) equipped with a field emission gun, a post-column Gatan imaging filter (model 2002), and a 2k × 2k post-GIF Gatan CCD camera (model 794). Micrographs were acquired at either 6,500× (electron dose: 1.64e^−^ Å^−2^ s^−1^) or 24,000× magnification (electron dose: 11.8e^−^ Å^−2^ s^−1^), using an acceleration voltage of 300kV in bright-field TEM mode with zero-loss energy filtering and 1s acquisition time.

### In vitro expression following transfection

Primary mouse bone-marrow-derived macrophages and primary human monocytes were seeded at 50,000 and 100,000 cells per well in 96-well plates, respectively. After overnight attachment, cells were transfected with aNPs containing IL-2 or mCherry mRNA (OZ Biosciences) at 0–4000 ng/well in a total volume of 200 μL. After 3 h, media were refreshed. Supernatants were collected at 24 h and human IL-2 quantified by ELISA (ELISA MAX Deluxe Set; BioLegend) per manufacturer’s protocol.

### Live cell imaging

Primary mouse bone marrow-derived macrophages were seeded at 50,000 cells per well in 96-well plates and allowed to attach overnight. Cells were transfected the next day with mCherry mRNA-loaded aNPs (5 μg/mL). After 1 h, media were refreshed, and plates were monitored using an IncuCyte S3 Live-Cell Analysis System (Sartorius) in a standard incubator (37 °C, 5% CO_2_). Phase-contrast and red-fluorescence images were acquired automatically every 2 h for 6 days. Image capture and analysis were performed using IncuCyte Live-Cell Imaging and Analysis Software (version v2025B). mCherry signal was quantified as Total Red Object Integrated Intensity (Relative Calibration Units × μm^2^ per image).

### HEK-Blue IL-2 reporter assay

Functional IL-2 activity in serum from NHPs treated with mRNA-aNPs was quantified using HEK-Blue IL-2 reporter cells (InvivoGen). These cells possess an intact STAT5 signaling pathway and harbor a SEAP reporter gene, producing SEAP in response to IL-2 stimulation. SEAP levels were measured using the QUANTI-Blue detection reagent (InvivoGen) according to the manufacturer’s instructions. Briefly, 20 μL of serum (undiluted or serially diluted) was combined with 180 μL of DMEM supplemented with 10% FBS and 1% penicillin-streptomycin containing 5 × 10^4^ reporter cells per well in a 96-well plate overnight. The assay was performed as previously described^35^.

### Preparing single-cell suspensions from the liver

Female C57BL/6J mice were euthanized and the largest liver lobe (≤1.2 g) was collected into 5 mL cold DMEM. Perfusion was performed on a gentleMACS Octo Dissociator with heaters using perfusion sleeves (Miltenyi Biotec). The lobe was fixed on a grid and processed with program “37C_m_LIPK_1”: priming (5 s), initial perfusion (30 s), three wash cycles (30 s), extended washing (13 min), equilibration (30 s), and enzymatic perfusion (10 min), with manual buffer exchanges at each step. Tissue was transferred to a gentleMACS C tube and dissociated using “LIPK_HR_1” (5 min). Cells were filtered through 70 μm MACS SmartStrainers, washed with flow buffer (PBS with 0.5% BSA and 2 mM EDTA), and centrifuged at 50 × g for 5 min at 4 °C.

### Preparing single-cell suspensions from tumor

Tumors (0.04–1 g) were dissociated using the Tumor Dissociation Kit (Miltenyi Biotec) following the manufacturer’s protocol. Fragments (2–4 mm) were enzymatically and mechanically dissociated in gentleMACS C tubes with enzyme mix (RPMI 1640 or DMEM, Enzyme D, Enzyme R, Enzyme A), processed on a gentleMACS Dissociator with heaters at 37 °C, filtered (70 μm), washed, and centrifuged. Red blood cells were lysed in 1× RBC lysis buffer (BioLegend 420302) for 7 min on ice, quenched with flow buffer, and centrifuged at 400 × g for 3 min at 4 °C.

### Preparing single-cell suspensions from blood, spleen, bone marrow, and lymph nodes

Spleens and lymph nodes were mechanically dissociated on 70 μm strainers using a syringe plunger and flow buffer. Bone marrow was collected by flushing femurs with 10 mL flow buffer and filtering through 70 μm strainers. Blood, spleen, and bone marrow cells were centrifuged at 400 × g for 3 min at 4 °C and supernatants aspirated. RBC lysis was performed with 1× buffer: blood (3 × 7 min on ice), spleen (1 × 7 min on ice), bone marrow (1 × 1 min at room temperature), followed by quenching with flow buffer and centrifugation at 400 × g for 3 min at 4 °C.

### Flow cytometry of mouse samples

The single cell suspensions were transferred to a Corning^®^ V-bottom 96-well plate and incubated with 50 μL viakrome 808 (#C36628, Beckman Coulter, diluted in PBS) for 5 minutes in the dark at room temperature. After washing, Fc receptors were blocked with anti-CD16/32 antibodies (Fc-block; 101302, BioLegend) for 5 minutes on ice. Cells were then stained with antibody cocktails diluted in Brilliant Stain Buffer (Invitrogen^™^, total volume 50 μL) and incubated on ice and in the dark for 30 minutes. All data were acquired using a 21-color CytoFLEX LX (Beckman Coulter). The gating strategies and antibodies used for myeloid, lymphoid, and liver cell populations in flow cytometry panels are listed in the Supplementary Information **(Supplementary Table 2–4, Supplementary Fig. 6).**

### In vivo IL-2 production following intravenous administration

aNPs encapsulating human IL-2 mRNA (OZ Biosciences) were administered intravenously to female C57BL/6J mice at 0.5 mg mRNA/kg in an injection volume of 100 μL. Organs (spleen, lymph nodes, bone marrow, liver) were harvested at 6, 12, 24, 48, and 72 h after injection and transferred to 2 mL BeadBug tubes containing 400 μL RIPA lysis buffer (Santa Cruz Biotechnology). Homogenization was performed with a BeadBug microtube homogenizer (Merck) for 1 min (spleen and lymph nodes), 40 s (liver), or 20 s (bone marrow). Lysates were centrifuged at 12,000 × g for 10 min and supernatants diluted for quantification of human IL-2 by ELISA (BioLegend) per manufacturer’s instructions. Serum was collected at the same time points and analyzed similarly. At 72 h, plasma clinical chemistry (AST, ALT, urea, creatinine) was measured by the Radboudumc Laboratorium voor Diagnostiek using a Roche/Hitachi cobas c 701/702 analyzer.

### Radiolabeling aNPs

dsRNA-azide (IDT technologies) and DFO-DBCO (Macrocyclics) were dissolved separately in Milli-Q water containing 5% (v/v) DMSO to 0.5 mg/mL. For DFO conjugation, dsRNA-azide (270 μL, 8 nmol) was reacted with DFO-DBCO (129 μL, 7.6 nmol) at room temperature for 16 h in the dark. The reaction was dialyzed (2 kDa MWCO; Sigma-Aldrich) against 1× PBS (1 L). Concentration was determined by NanoDrop and integrity verified by 1% agarose gel electrophoresis. Products were lyophilized and stored at −20 °C.

For radiolabeling, ^89^Zr oxalate in 1 M oxalic acid was neutralized to pH 6.8–7.4 with 1 M sodium carbonate (total volume < 25 μL) and added to DFO-dsRNA (0.5 mL in water). The mixture was incubated at 37 °C for 60 min at 300 rpm. Radiochemical conversion was confirmed by radio-TLC and yields exceeded 80%. ^89^Zr-labeled RNAs were co-formulated with unlabeled mRNA at a 50:50 (w/w) dsRNA:mRNA input to generate aNP-^89^Zr-dsRNA/mRNA. Final formulations were characterized by cryo-EM (morphology), DLS (size/PDI), and RiboGreen (encapsulation).

The dsRNA-azide sequence:

5 -rArCrCrCrUrGrArArGrUrUrCrArUrCrUrGrCrArCrCrArCCG/3AzideN/−3’.

5’-rCrGrGrUrGrGrUrGrGrArGrArUrGrArArCrUrUrCrArGrGrGrUrCrA-3’.

### Biodistribution and pharmacokinetics of ^89^Zr-aNPs

Female C57BL/6J mice were intravenously administered with aNP-^89^Zr-dsRNA/mRNA (8 ^89^Zr correlating to 0.16–0.5 mg RNA/kg, specific activity of 1–5 mCi/mg) in 100 μL PBS via tail-vein injection. At 1, 5, 15, 30 minutes, 1, 2, 4, and 24 hours, blood was collected via tail puncture to determine the pharmacokinetic profile and to calculate the weighted weighted blood half-life. At 24 hours after injection, mice were euthanized, perfused with PBS, and tissues (bone marrow, spleen, heart, lung, muscle, lymph nodes, blood, liver, and kidneys) were collected and weighed. Next, the emitted γ radiation was measured on a gamma counter (2480 WIZARD2 Automatic Gamma Counter, PerkinElmer). Radioactivity values were corrected for decay and normalized to tissue weight to express radioactivity concentration as percentage of injected dose per gram (%ID/g).

### Radiolabeling CD8 nanobody probe and biodistribution

The PEG20-CD8-DFO probe (1.05 mg/mL) was radiolabeled with ^89^Zr, achieving a specific activity of 5.4 mCi/mg. A dose of 3 μg of the PEG20-CD8-DFO-labeled probe (16 μCi per mouse) was administered via injection. At 24 hours post-injection, mice were euthanized and perfused with PBS. Subsequently, tissues including bone marrow, spleen, heart, lung, muscle, lymph nodes, blood, liver, kidneys, and tumor were harvested, weighed, and analyzed. The emitted gamma radiation from each tissue sample was quantified using a gamma counter (2480 WIZARD2 Automatic Gamma Counter, PerkinElmer). Radioactivity measurements were decay-corrected and normalized to tissue weight, and results were expressed as the percentage of injected dose per gram of tissue (%ID/g).

### PET/CT imaging acquisition and analysis

PET imaging was executed 24 hours after aNP-^89^Zr-dsRNA/mRNA or ^89^Zr-PEG20-CD8-DFO injection using an IRIS PET/CT (Inviscan). Mice were anesthetized with a gas mixture of 2 % isoflurane and 5 % oxygen. A 10-minute static whole-body PET scan was conducted using an energy window between 250 – 750 keV followed by a 40 second whole-body CT scan (energy 80 kV, 0.9 mA, 576 projections, voxel size 160 μm). PET images were reconstructed with the 3D Ordered Subsets Expectation Maximization (3D-OSEM- MC) algorithm (8 subsets and 8 iterations) using decay, random, and dead-time correction. All PET and CT data were processed using OsiriX Medical Imaging software (version 13.0.3).

### B16F10 tumor inoculation and treatment regimen

Female C57BL/6J mice were inoculated with 1×10^5^ B16F10 (ATCC) cells in 100 μL PBS via subcutaneous injection in the right flank on day −7. Mice without palpable tumors on day 0 were excluded from the experiment, and the included mice were allocated to treatment and control groups. Treatment for all experiments consisted of intravenous administration (1 × 0.5 mg/kg in 14 days, 1×0.16 mg/kg in 14 days, 4×0.125 mg/kg 4 days apart, 4×0.04 mg/kg 4 days apart) of aNP-HMJ1 containing IL-2 mRNA or 4×0.125 mg/kg 4 days apart of aNP-HMJ1 containing IFN-y-encoding mRNA. Treatment started on day 0 and lasted until day 12. Log_10_ transformation of tumor sizes and the growth rate calculations were based on Hather G, et. al. (2014)^36^. Mice were euthanized in accordance with Dutch animal welfare regulations once they reached the predefined humane endpoint criteria, which included a tumor volume of 1500 mm^3^ or greater, the occurrence of tumor ulceration, or observable signs of distress.

### MC38 tumor inoculation and treatment regimen

Female C57BL/6J mice were inoculated with 1×10^6^ MC38 (Merck) cells in 100 μL PBS via subcutaneous injection in the right flank on day −10. Mice with tumor sizes <14.14 mm^3^ and >100 mm^3^ on day 0 were excluded from the experiment, and the included mice were allocated to treatment and control groups. Treatment for all experiments consisted of intravenous administration (1×0.5 mg/kg in 14 days, 1×0.16 mg/kg in 14 days, 4×0.125 mg/kg 4 days apart, 4×0.04 mg/kg 4 days apart) of aNP-ALC0315, aNP-HMJ1, or aNP-HMJ2 containing human IL-2 mRNA. Treatment started on day 0 and lasted until day 12. Mice were euthanized in accordance with Dutch animal welfare regulations once they reached the predefined humane endpoint criteria, which included a tumor volume of 1500 mm^3^ or greater, the occurrence of tumor ulceration, or observable signs of distress.

### Non-human primate studies

Two adult male cynomolgus macaques (Macaca fascicularis), weighing 5.98 kg and 9.6 kg, were included in the non-human primate studies. Animals were housed in pairs whenever possible under climate-controlled conditions with a 12-hour light/dark cycle. They were provided water ad libitum and fed Teklad Global 20% Protein Primate Diet.

### In vivo PET/MR of non-human primates

PET and MR imaging were performed on a combined 3T hybrid PET/MRI system (Biograph mMR, Siemens Healthineers). After an overnight fast, animals were anesthetized with ketamine (5.0 mg/kg) and dexmedetomidine (0.0075–0.015 mg/kg), and blood samples were obtained from the femoral vein. While positioned on the scanner, animals received intravenous injections of ^89^Zr-labeled mRNA-aNPs.

#### PET acquisition:

Dynamic PET was acquired over the thorax using a single bed position for 60 minutes immediately following tracer injection. Subsequently, static whole-body PET scans were obtained at 1 and 24 hours post-injection, covering the cranium to pelvis in four consecutive bed positions. PET data were collected for 20 minutes per bed with a reconstructed voxel size of 2.3 × 2.3 × 5.0 mm. Reconstructions were performed online using an ordered subset expectation maximization (OSEM) algorithm with point spread function (PSF) correction, 3 iterations, and 21 subsets. Attenuation correction employed both Dixon-based MR attenuation correction (MRAC_DIXON; voxel size 2.6 × 2.6 × 3.1 mm) and a high-resolution CAIPI Dixon sequence (MRAC_CAIPI_HiRes; voxel size 1.3 × 1.3 × 3.0 mm).

#### MR acquisition:

PET images were fused with simultaneously acquired MR sequences. For anatomical reference, a T1-weighted 3D turbo spin echo sequence (T1-SPACE) was acquired in coronal orientation with 1.0 mm slice thickness, 224 slices, field of view 300 × 267 mm, in-plane resolution 1.17 mm × 1.17 mm^2^, TR 1000 ms, TE 78 ms, turbo factor 51, bandwidth 592 Hz/pixel, and acquisition time 14:28 minutes per bed. Additional sequences included coronal HASTE (voxel size 1.6 mm × 1.6 mm^2^ TR 1000 ms, TE 40 ms, turbo factor 144, bandwidth 704 Hz/pixel, slice thickness 5 mm, SPAIR fat saturation), PD-SPACE multi-bed imaging (voxel size 1.17 mm × 1.17 mm × 1.0 mm, TR 1000 ms, TE 78 ms, SPAIR fat saturation), and high-resolution T2-SPACE with respiratory triggering (1.0 mm isotropic resolution, TR 2400 ms, TE 698 ms, turbo factor 180, bandwidth 352 Hz/pixel, GRAPPA acceleration factor 3).

### PET/MR image analysis

Volumes of interest (VOIs) were manually delineated for the spleen, vertebral bone marrow, lymph nodes and skeletal muscle using the MR images. The mean standardized uptake value (SUV_mean_) was extracted from these VOIs using the 24-hour static PET scan. For pharmacokinetic analysis, the 60-minute dynamic PET scans were reconstructed in 6 frames of 10 s, 4 frames of 30 s, 1 frame of 60 sc, 3 frames of 120 s, 4 frames of 300 s, and 3 frames of 600 s. Blood pool VOIs (~ 1cm^3^) were drawn in center of the left ventricular cavity. Maximum SUVs (SUV_max_) were calculated for all the frames of the dynamic scan as well as for the 24-hour static PET scan and fitted using a biexponential decay function.

### Non-human primate flow cytometry

Cynomolgus macaque blood samples (700 μL from EDTA tube) were lysed three times using RBC lysis (420301, BioLegend) for 7 minutes on ice. Cells were stained in FACS tubes with 250× diluted Zombie UV dye (423108, BioLegend) for viability and washed once with flow buffer. Fc-block treatment (42230, BioLegend, 25× dilution) was performed on ice for 10 minutes, followed by another wash. Cells were incubated with the antibody cocktail (NHP cell populations flow cytometry panel) in Brilliant Stain Buffer (Invitrogen) for 30 minutes on ice and in the dark, rewashed, and resuspended in flow buffer prior to analysis. Data acquisition and compensation were performed on a Cytek Aurora cytometer. The gating strategy and antibodies used to identify the immune cell populations in flow cytometry panels are listed in the Supplementary Information **(Supplementary Table 5, Supplementary Fig. 7).**

### Data representation and statistical analysis

All data values are presented as mean ± s.d. or mean ± s.e. for tumor growth graphs. Samples were excluded from the analysis if the cell count from a tissue was insufficient to ensure reliable flow cytometry data. Data was analyzed with FlowJo version 10.10.0 (BD Biosciences) or OMIQ (Dotmatics). Statistical analyses were performed using GraphPad Prism 10.0 by one-way or two-way analysis of variance (ANOVA) with a Bonferroni post-hoc test. Survival outcomes were determined according to predefined humane endpoint criteria specific to each tumor model, and the resulting data were analyzed using the log-rank (Mantel-Cox) test. The difference was considered significant if *P* < 0.05. Levels of significance are indicated as follows: *P < 0.05; ** *P* < 0.01; *** *P* < 0.001; **** *P* < 0.0001.

## Supplementary Material

Supplementary Files

This is a list of supplementary files associated with this preprint. Click to download.

• SupplementaryInformationAnbergenetal.pdf

## Data Availability

All relevant data are provided in this Article and its Supplementary Information is available for this paper.

## References

[1] Kirkwood J. M. Next Generation of Immunotherapy for Melanoma. Journal of Clinical Oncology 26, 3445–3455 (2008). 18612161 10.1200/JCO.2007.14.6423

[2] Lipton J. H. Phase II, randomized, multicenter, comparative study of peginterferon-α−2a (40 kD) (Pegasys^®^) versus interferon α−2a (Roferon^®^-A) in patients with treatment-naïve, chronic-phase chronic myelogenous leukemia. Leuk Lymphoma 48, 497–505 (2007). 17454589 10.1080/10428190601175393

[3] Vachhani P. Interferons in the treatment of myeloproliferative neoplasms. Ther Adv Hematol 15, (2024). 10.1177/20406207241229588 PMC10878223 38380373

[4] Eggermont A. M. Adjuvant therapy with pegylated interferon alfa-2b versus observation alone in resected stage III melanoma: final results of EORTC 18991, a randomised phase III trial. The Lancet 372, 117–126 (2008). 10.1016/S0140-6736(08)61033-8 18620949

[5] Eigentler T. K. Adjuvant treatment with pegylated interferon α−2a versus low-dose interferon α−2a in patients with high-risk melanoma: A randomized phase III DeCOG trial. Annals of Oncology 27, 1625–1632 (2016). 27287206 10.1093/annonc/mdw225

[6] Nilsson M. S. Immunotherapy with HDC/IL-2 may be clinically efficacious in acute myeloid leukemia of normal karyotype. Hum Vaccin Immunother 16, 109–111 (2020). 31242079 10.1080/21645515.2019.1636598 PMC7012093

[7] Dutcher J. P. High dose interleukin-2 (Aldesleukin) - expert consensus on best management practices-2014. J Immunother Cancer 2, (2014). 10.1186/s40425-014-0026-0 PMC6889624 31546315

[8] Rosenberg S. A. IL-2: The First Effective Immunotherapy for Human Cancer. J Immunol 192, 5451 (2014). 24907378 10.4049/jimmunol.1490019 PMC6293462

[9] Zhou Y. Interleukin 15 in Cell-Based Cancer Immunotherapy. Int J Mol Sci 23, 7311 (2022). 35806311 10.3390/ijms23137311 PMC9266896

[10] Nastala C., Edington H D. & McKinney T G. Recombinant IL-12 administration induces tumor regression in association with IFN-gamma production - PubMed. Journal of Immunology 153, 1697–706 (1994). 7913943

[11] Vadhan-Raj S. Phase I trial of recombinant interferon gamma in cancer patients. Journal of Clinical Oncology 4, 137–146 (1986). 3080551 10.1200/JCO.1986.4.2.137

[12] Study Details | NCT00089193 | Vaccine Therapy With or Without Sargramostim in Treating Patients With Stage IIB, Stage IIC, Stage III, or Stage IV Melanoma | ClinicalTrials.gov. https://www.clinicaltrials.gov/study/NCT00089193.

[13] Clancy-Thompson E. Peptide vaccination in Montanide adjuvant induces and GM-CSF increases CXCR3 and cutaneous lymphocyte antigen expression by tumor antigen-specific CD8 T cells. Cancer Immunol Res 1, 332–339 (2013). 24377099 10.1158/2326-6066.CIR-13-0084 PMC3873151

[14] Charych D. H. NKTR-214, an Engineered Cytokine with Biased IL2 Receptor Binding, Increased Tumor Exposure, and Marked Efficacy in Mouse Tumor Models. Clinical Cancer Research 22, 680–690 (2016). 26832745 10.1158/1078-0432.CCR-15-1631

[15] Silver A. B. An engineered immunocytokine with collagen affinity improves the tumor bioavailability, tolerability, and therapeutic efficacy of IL-2. Cell Rep Med 4, (2023). 10.1016/j.xcrm.2023.101289 PMC10694763 37992685

[16] Beck J. D. Long-lasting mRNA-encoded interleukin-2 restores CD8+ T cell neoantigen immunity in MHC class I-deficient cancers. Cancer Cell 42, 568–582.e11 (2024). 38490213 10.1016/j.ccell.2024.02.013

[17] Deckers J. Engineering cytokine therapeutics. Nature Reviews Bioengineering 1, 286–303 (2023). 10.1038/s44222-023-00030-y PMC9933837 37064653

[18] Hofstraat S. R. J. Nature-inspired platform nanotechnology for RNA delivery to myeloid cells and their bone marrow progenitors. Nat Nanotechnol 20, 532–542 (2025). 39900620 10.1038/s41565-024-01847-3 PMC12014499

[19] Hou X., Zaks T., Langer R. & Dong Y. Lipid nanoparticles for mRNA delivery. Nat Rev Mater 6, 1078–1094 (2021). 34394960 10.1038/s41578-021-00358-0 PMC8353930

[20] Leal J. M. Innate cell microenvironments in lymph nodes shape the generation of T cell responses during type I inflammation. Sci Immunol 6, (2021). 10.1126/sciimmunol.abb9435 PMC8274717 33579750

[21] de Kruijf E. J. F. M., Fibbe W. E. & van Pel M. Cytokine-induced hematopoietic stem and progenitor cell mobilization: unraveling interactions between stem cells and their niche. Ann N Y Acad Sci 1466, 24–38 (2020). 31006885 10.1111/nyas.14059 PMC7217176

[22] Semaeva E. Access to the spleen microenvironment through lymph shows local cytokine production, increased cell flux, and altered signaling of immune cells during lipopolysaccharide-induced acute inflammation. J Immunol 184, 4547–4556 (2010). 20237290 10.4049/jimmunol.0902049

[23] Trines M. M. Dendrimers Improve Apolipoprotein Nanoparticle mRNA Delivery to Immune Cells. Advanced Materials 2025, e04830 (2025). 10.1002/adma.202504830 PMC12801360 40937965

[24] Ross S. H. & Cantrell D. A. Signaling and Function of Interleukin-2 in T Lymphocytes. Annu Rev Immunol 36, 411 (2018). 29677473 10.1146/annurev-immunol-042617-053352 PMC6472684

[25] Hwang J. R., Byeon Y., Kim D. & Park S. G. Recent insights of T cell receptor-mediated signaling pathways for T cell activation and development. Experimental & Molecular Medicine 2020 52:5 52, 750–761 (2020). 10.1038/s12276-020-0435-8 PMC7272404 32439954

[26] Waldman A. D., Fritz J. M. & Lenardo M. J. A guide to cancer immunotherapy: from T cell basic science to clinical practice. Nat Rev Immunol 20, 651–668 (2020). 32433532 10.1038/s41577-020-0306-5 PMC7238960

[27] Mosmann T. R. Species-specificity of T cell stimulating activities of IL 2 and BSF-1 (IL 4): comparison of normal and recombinant, mouse and human IL 2 and BSF-1 (IL 4). Journal of immunology 138 6, 1813–6 (1987). 3493289

[28] Spranger S., Bao R. & Gajewski T. F. Melanoma-intrinsic β-catenin signalling prevents anti-tumour immunity. Nature 523, 231–235 (2015). 25970248 10.1038/nature14404

[29] Teunissen A. J. P. Employing nanobodies for immune landscape profiling by PET imaging in mice. STAR Protoc 2, 100434 (2021). 33899016 10.1016/j.xpro.2021.100434 PMC8056265

[30] IL-2 & IL-15 Reporter Cells | CD25, CD122, and CD132 | InvivoGen. https://www.invivogen.com/hek-blue-il2.

[31] O Brien Laramy M. Chemistry, manufacturing and controls strategies for using novel excipients in lipid nanoparticles. Nat Nanotechnol 20, 331–344 (2025). 39821140 10.1038/s41565-024-01833-9

[32] Huang X., Ma Y., Ma G. & Xia Y. Unlocking the Therapeutic Applicability of LNP-mRNA: Chemistry, Formulation, and Clinical Strategies. Research 7, (2024). 10.34133/research.0370 PMC11185168 38894715

[33] Yang K. Coordinating interleukin-2 encoding circRNA with immunomodulatory lipid nanoparticles to potentiate cancer immunotherapy. Sci Adv 11, (2025). 10.1126/sciadv.adn7256 PMC11864171 40009662

[34] Jeffs L. B. A scalable, extrusion-free method for efficient liposomal encapsulation of plasmid DNA. Pharm Res 22, 362–372 (2005). 15835741 10.1007/s11095-004-1873-z

[35] Schrijver D. P. Resolving sepsis-induced immunoparalysis via trained immunity by targeting interleukin-4 to myeloid cells. Nature Biomedical Engineering 2023 7:9 7, 1097–1112 (2023). 10.1038/s41551-023-01050-0 PMC10504080 37291433

[36] Hather G. Growth Rate Analysis and Efficient Experimental Design for Tumor Xenograft Studies. Cancer Inform 13, 65 (2014). 25574127 10.4137/CIN.S13974 PMC4264612

